# Electrical response of retinal ganglion cells in an *N*-methyl-*N*-nitrosourea-induced retinal degeneration porcine model

**DOI:** 10.1038/s41598-021-03439-w

**Published:** 2021-12-17

**Authors:** Seongkwang Cha, Kwang-Eon Choi, Jungryul Ahn, Minsu Yoo, Yurim Jeong, Seong-Woo Kim, Yong Sook Goo

**Affiliations:** 1 Department of Physiology, Chungbuk National University School of Medicine, Cheongju, 28644 Korea; 2 Department of Ophthalmology, Korea University College of Medicine, Seoul, 08308 Korea

**Keywords:** Retina, Neurophysiology, Neurodegeneration, Retinal diseases

## Abstract

Retinal prosthesis is regarded as the treatment for vision restoration in the blind with retinal degeneration (RD) due to the loss of photoreceptors. A strategy for retinal prosthesis is to electrically activate surviving neurons. The retina’s response to electrical stimulation in a larger RD model has not been studied yet. Therefore, in this study, we investigated electrically evoked retinal responses in a previously validated *N*-methyl-*N*-nitrosourea (MNU)-induced porcine RD model. Electrically evoked responses were evaluated based on the number of retinal ganglion cell (RGC) spikes via multichannel recordings. Stimulation pulses were applied to degenerative and wild-type retinas with pulse modulation. Compared to wild-type retinas, degenerative retinas showed higher threshold values of pulse amplitude and pulse duration. The rate of increase in the number of RGC spikes relative to stimulus intensity was lower in degenerative retinas than in normal retinas*.* In severely degenerated retinas, few RGCs showed electrically evoked spikes. Our results suggest that the degenerative porcine retina requires a higher charge than the normal porcine retina. In the early stage of RD, it is easier to induce RGC spikes through electrical stimulation using retinal prosthesis; however, when the degeneration is severe, there may be difficulty recovering patient vision.

## Introduction

Retinal degenerative diseases, such as retinitis pigmentosa (RP) and age-related macular degeneration (AMD), are major causes of blindness in adults^1,2^. Such retinal degeneration (RD) occurs worldwide, wherein their incidence increases with age^3^. RD begins with a gradual loss of photoreceptors. However, other retinal neurons, such as bipolar cells (BCs) or retinal ganglion cells (RGCs), still survive and maintain their functions even after the complete death of photoreceptors^4–7^. Therefore, in the case of patients with RD, it is still possible to send visual information to the brain using surviving retinal neurons.

Given these possibilities, retinal prostheses have been developed to restore vision in patients with RD. These devices are designed to elicit spikes of RGCs by applying electrical stimuli to surviving neurons to bypass dead photoreceptors^8–10^. Thus, understanding how normal and degenerated retinal networks respond to electrical stimulation is one of the most important prerequisites for the successful development of a retinal prosthesis for the blind.

Several studies have been conducted to establish optimal electrical stimulation parameters^11–20^. Particularly, it has been reported that the biphasic pulse has an advantage over the monophasic pulse in reducing damage in retinal tissues^21^. Considering biphasic pulses, anodic or cathodic phase-first biphasic pulses show different efficacies depending on the type of prosthesis. Epiretinal stimulation exhibits better efficacy when cathodic phase-first biphasic pulses are used, whereas subretinal stimulation exhibits better efficacy in case of anodic phase-first biphasic pulses^22,23^. However, as these studies examined only lower mammals, such as mice, rats, and rabbits, it is difficult to extrapolate their results to patients with RD.

Although the basic structure of the retinal information flow (from the photoreceptor, BC, to RGC) and feedback modulation [from horizontal cells (HCs) and amacrine cells (ACs)] are mostly similar among different species, considerable histological differences, such as in the distribution of neurons, the thickness of the retina, and the presence of fovea exist among mammals^24,25^. In addition, visual information processing in the retinal network to a visual stimulus also differs among species^26^. However, studies that directly show the differences in RGC responses to electrical stimulation among different species are currently scanty. Although Sekirnjack et al. reported no remarkable differences in RGC responses among monkeys, guinea pigs, and rats under the same stimulation conditions^19^, further research is required in this regard. Because their results were analyzed based only on the direct RGC responses with a short latency (less than 20 ms) without considering indirect responses of RGCs, investigating the indirect responses of RGCs through retinal synapses among different mammals is indeed crucial.

More importantly, it is necessary to optimize parameters for electrical stimulation of RGCs using models with larger mammals that are evolutionarily closer to humans. We chose the porcine model because of several similarities in the eyes of pigs and humans, not only in the size of the eyeball but also in its composition and the shape of the retina^27^. Previously, we developed an *N*-methyl-*N*-nitrosourea (MNU)-induced porcine RD model, and showed that the porcine model is a good animal model for patients with RP^28^. In this study, we investigated the correlation between the severity of RD and electrically elicited RGC responses using the same porcine model.

## Results

### Classifying in-vitro RD severity

**Figure 1:**
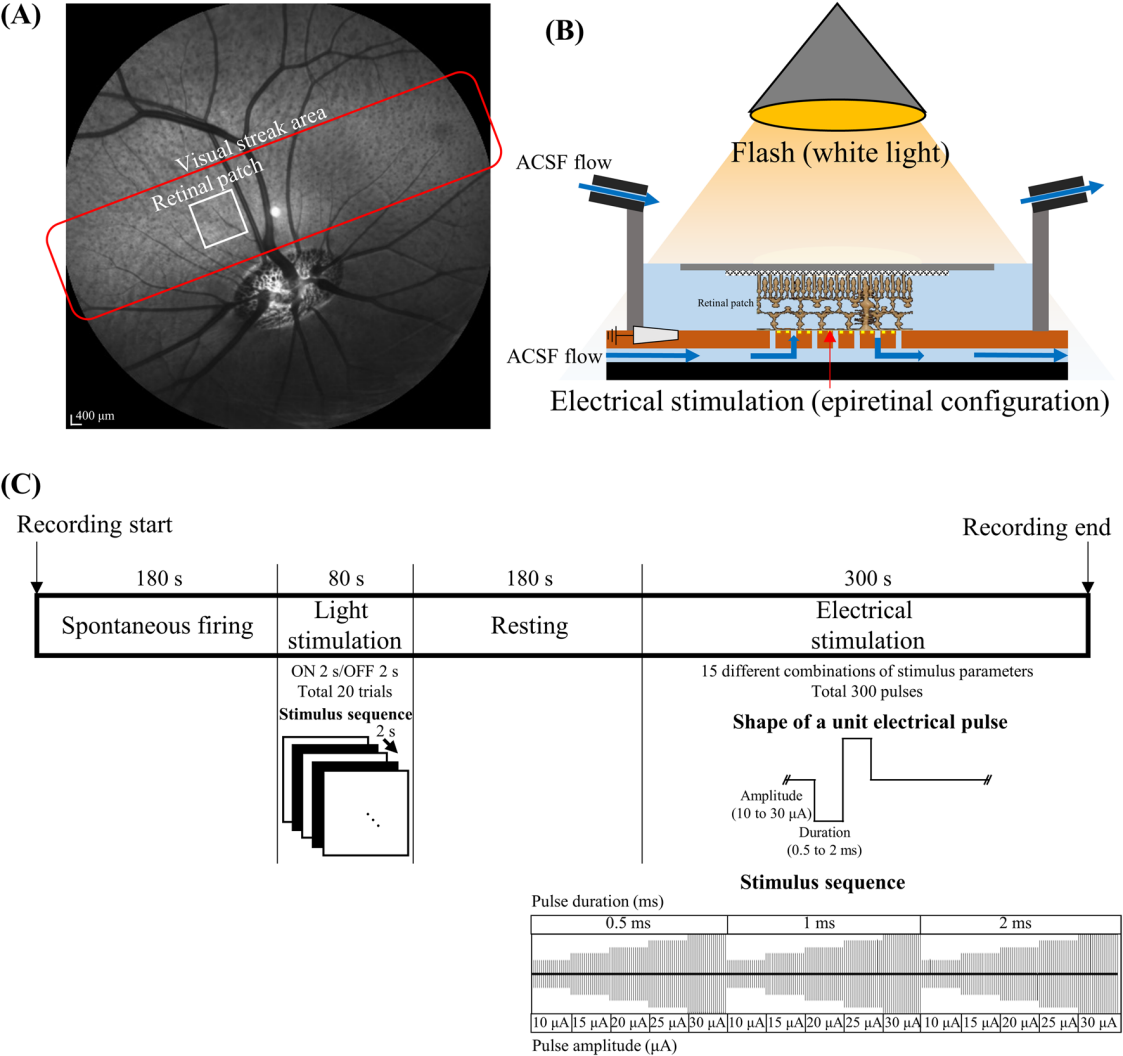
Experimental setup and protocol for in-vitro multi-electrode array (MEA) recording. (**A**) The retinal patch was isolated from the visual streak area. The MEA covers 2 mm × 2 mm of the local area in the entire retina (white box in the visual streak area). (**B**) The isolated retinal patch was mounted on the MEA with the RGC layer facing the electrode array. (**C**) The experimental protocol.

In our previous study, we reported the reduced light responsiveness in MNU-induced porcine RD model using the full-field electroretinogram (ffERG) recording^28^, wherein ERG recording represented the overall average responses of photoreceptors (a-wave) and BCs (b-wave). In this study, we focused on the responses of individual RGCs using an in-vitro multi-electrode array (MEA) recording setup to identify changes in light responsiveness in local areas (2 mm × 2 mm) of the retinal tissue, not the whole retina (Fig. 1A).

**Figure 2:**
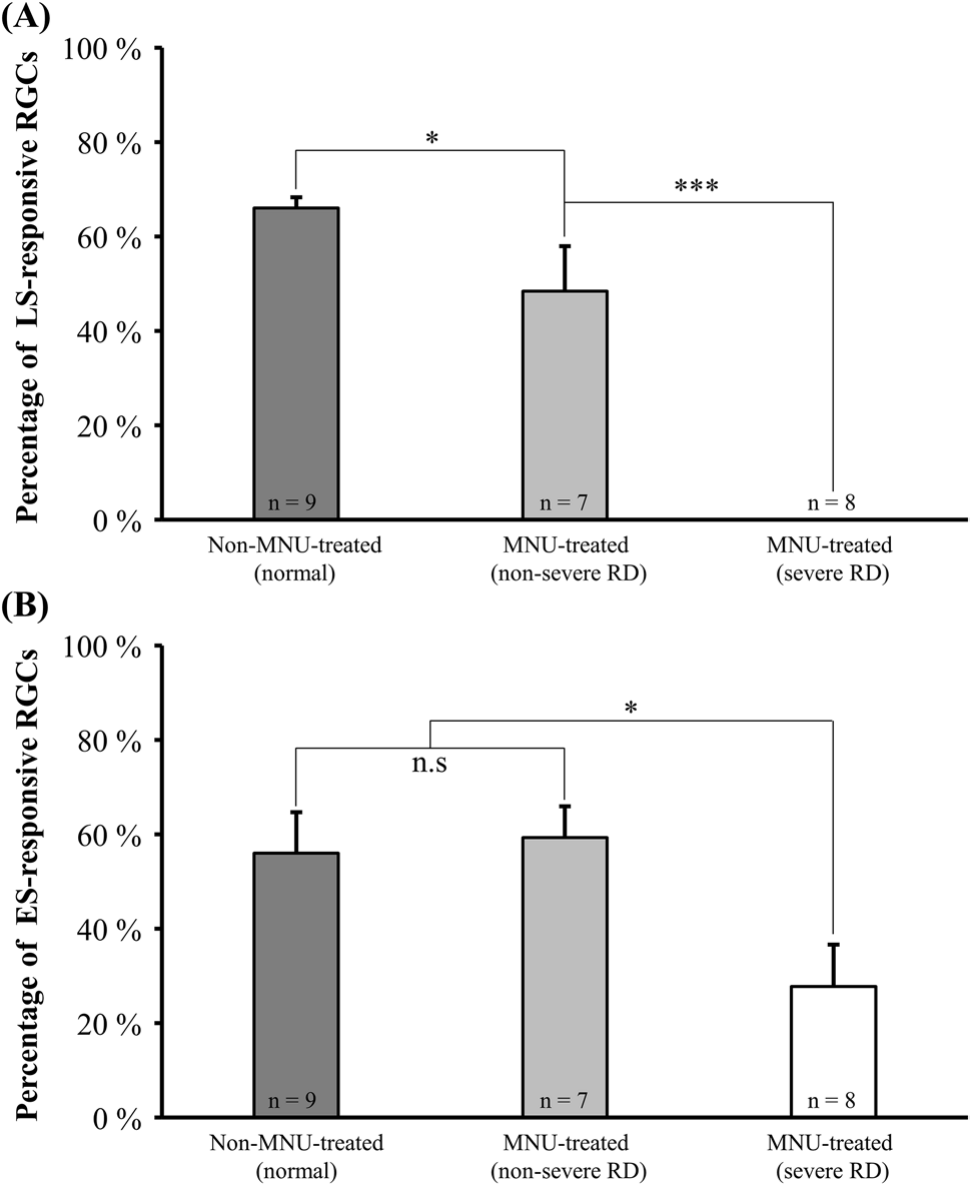
Differences in the number of RGCs that respond to light or electrical stimulation based on presence or absence of MNU treatment. (**A**) Average population of light stimulus (LS)-responsive RGCs in the non-MNU-treated (normal), MNU-treated (non-severe RD), and MNU-treated (severe RD) groups. Error bars represent the standard error of the mean (SEM). (**B**) Average population of electrical stimulus (ES)-responsive RGCs in the three groups. Statistical difference among the three groups analyzed using ANOVA, post-hoc test, and Tukey’s HSD test (^n.s^p > 0.05, *p < 0.05, and ***p < 0.001).

To classify the levels of severity of RD, we calculated the percentage of changes in light stimulus (LS) responses of all RGCs in all retinal patches recorded. LS-responsive RGCs showed three types of responses: ON, OFF, and ON/OFF (see Supplementary Fig. S1). As a control group, the non-MNU-treated (normal) group showed 66.1 ± 2.2% (mean ± SEM) LS-responsiveness (n = 9 retinal patches) (Fig. 2A). We classified MNU-treated retinal patches (n = 15) into two groups, non-severe and severe RD, based on the existence and non-existence of LS-responsive RGCs, respectively. If the tested retinal patch did not respond to LS, we classified the retinal patch as a severe RD group. The other retinal patches were classified as the non-severe RD group. The average percentage of LS-responsiveness in the non-severe RD group was 48.4 ± 2.2% (n = 7 retinal patches).

### Responsiveness to electrical stimulation of RGCs according to the degree of RD severity

We previously found that even after the photoreceptor layer degenerated, RGCs remained intact^28^. Therefore, in the current study, we speculated that RGCs would be differentially responsive to electrical stimuli (ES) with varied current pulses (duration: 0.5, 1, and 2 ms; amplitude: 10, 15, 20, 25, and 30 μA). Figure 2B shows the percentage of ES-responsive RGCs in the three RD groups at different severity of degeneration at the strongest level of stimulus (2 ms pulse duration and 30 μA pulse amplitude). No significant differences were observed in the percentage of ES-responsive RGCs between the normal and non-severe RD groups (56.1 ± 8.7% and 59.3 ± 6.6%, respectively). However, only about half of the average percentage of ES-responsive RGCs (27.8 ± 8.9%) were seen in the severe RD group.

### MNU-treated RD retina is well modulated by the current stimulation protocol, similar to the normal retina

**Figure 3:**
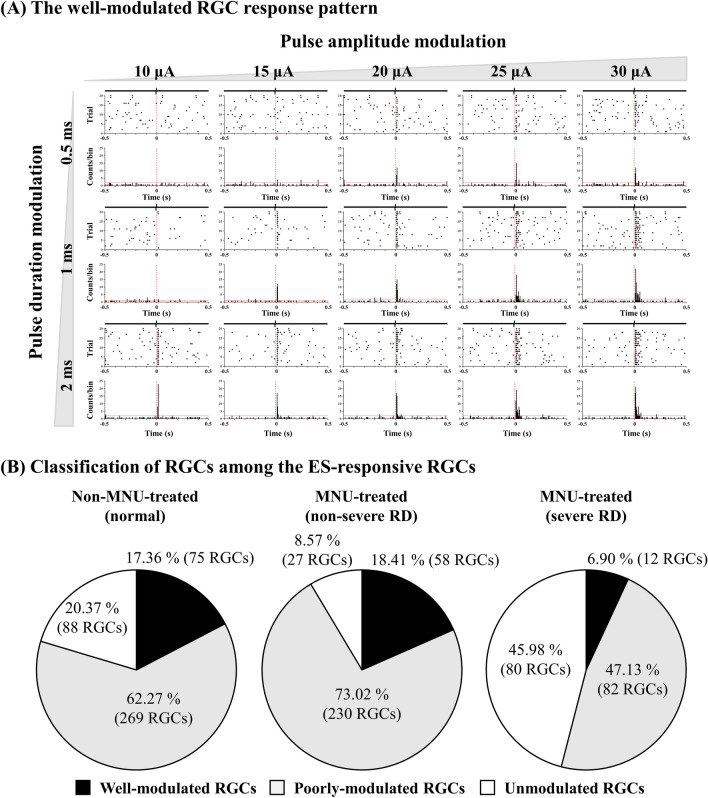
Response pattern of RGCs to electrical pulse amplitude and duration modulation. (**A**) A typical well-modulated RGC response pattern in raster presentation (upper row) and post-stimulus time as histograms (PSTH, lower row). The pulse amplitude of electrical stimulation was modulated into five steps from 10 to 30 μA. With each pulse amplitude, the pulse duration of electrical stimulation was also modulated into three steps from 0.5 to 2 ms. The time bin of PSTH was 20 ms. The red vertical dashed lines in the raster plot and PSTH indicate electrical stimulus onset. The red horizontal lines in the PSTH indicate 99% confidence level. (**B**) RGCs classified as well-modulated, poorly modulated, and unmodulated based on their response pattern to electrical stimulation modulation.

In our previous study, we observed that RGCs in general do not respond proportionally to changes in the strength of electrical stimulation in mice^29^. We also found such non-proportional RGC responses in pigs to various levels of pulse amplitude modulation (PAM) and pulse duration modulation (PDM). Therefore, in this study, we selected only well-modulated RGCs that showed a proportional response curve drawn from the levels of RGC responses to the various combinations of electrical stimulation with different durations (0.5, 1, and 2 ms) and amplitudes (10, 15, 20, 25, and 30 μA). The ES-responsive RGCs of each group are described in Fig. 2B. Briefly, only 17.36% (75/432), 18.41% (58/315), and 6.90% (12/174) of RGCs were well-modulated throughout all three PAM and five PDM protocols in the normal, non-severe, and severe RD groups, respectively (Fig. 3).

**Figure 4:**
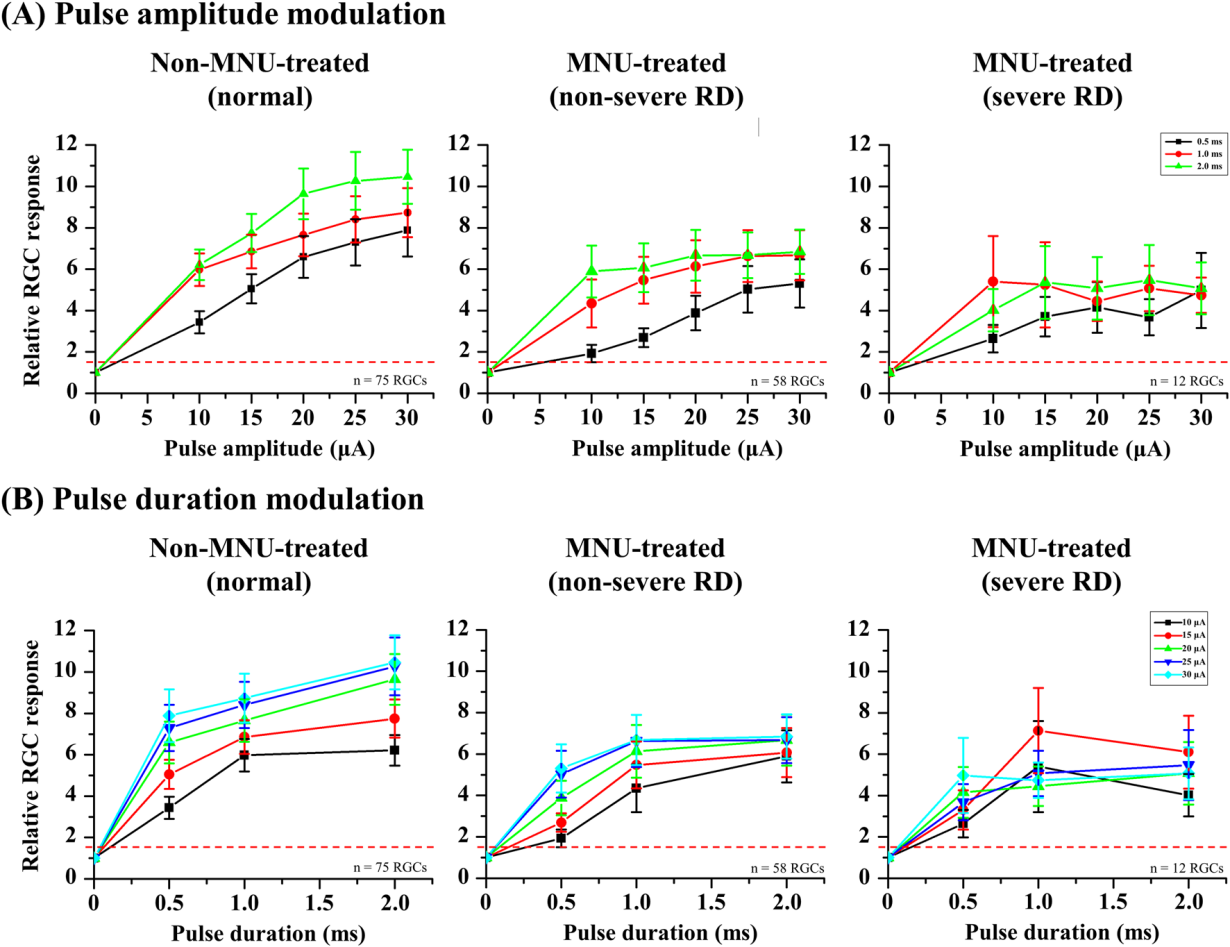
ES-responsiveness of well-modulated RGCs to pulse amplitude modulation (PAM) and pulse duration modulation (PDM). (**A**) Average relative RGC response plotted against pulse amplitude from 0.5 to 2 ms fixed duration for non-MNU**-**treated (left), MNU**-**treated non-severe RD (middle), and MNU**-**treated severe RD (right) groups. Error bars represent the SEM. The threshold level is plotted as red dashed line at 1.5 times. (**B**) Mean evoked RGC spike number plotted against pulse duration from 10 to 30 μA fixed amplitude.

Figure 4 shows the curves of the average RGC responses of well-modulated RGCs in three different severities of the RD groups. In the normal group, the RGC responses tended to increase proportionally with an increase in pulse amplitude and duration. However, MNU-treated groups showed saturated RGC responses even at the stimulation level that induced a proportional increase in the normal group. In the non-severe RD group, the RGC response curve started to saturate from a pulse amplitude of approximately 20 μA with a fixed pulse duration of 1 ms. In addition, the RGC responses were rarely modulated by a 2-ms duration fixed pulse. In the severe RD group, the RGC responses were rarely modulated by fixed 1- or 2-ms duration pulses. Similar to the results of the PAM experiment, the RGC responses tended to increase proportionally with an increase in pulse duration in the normal and non-severe RD groups. However, in the severe RD group, RGC responses were rarely modulated by all the PDM protocols.

### Thresholds of pulse amplitude and pulse duration differ according to the degree of RD severity

**Figure 5:**
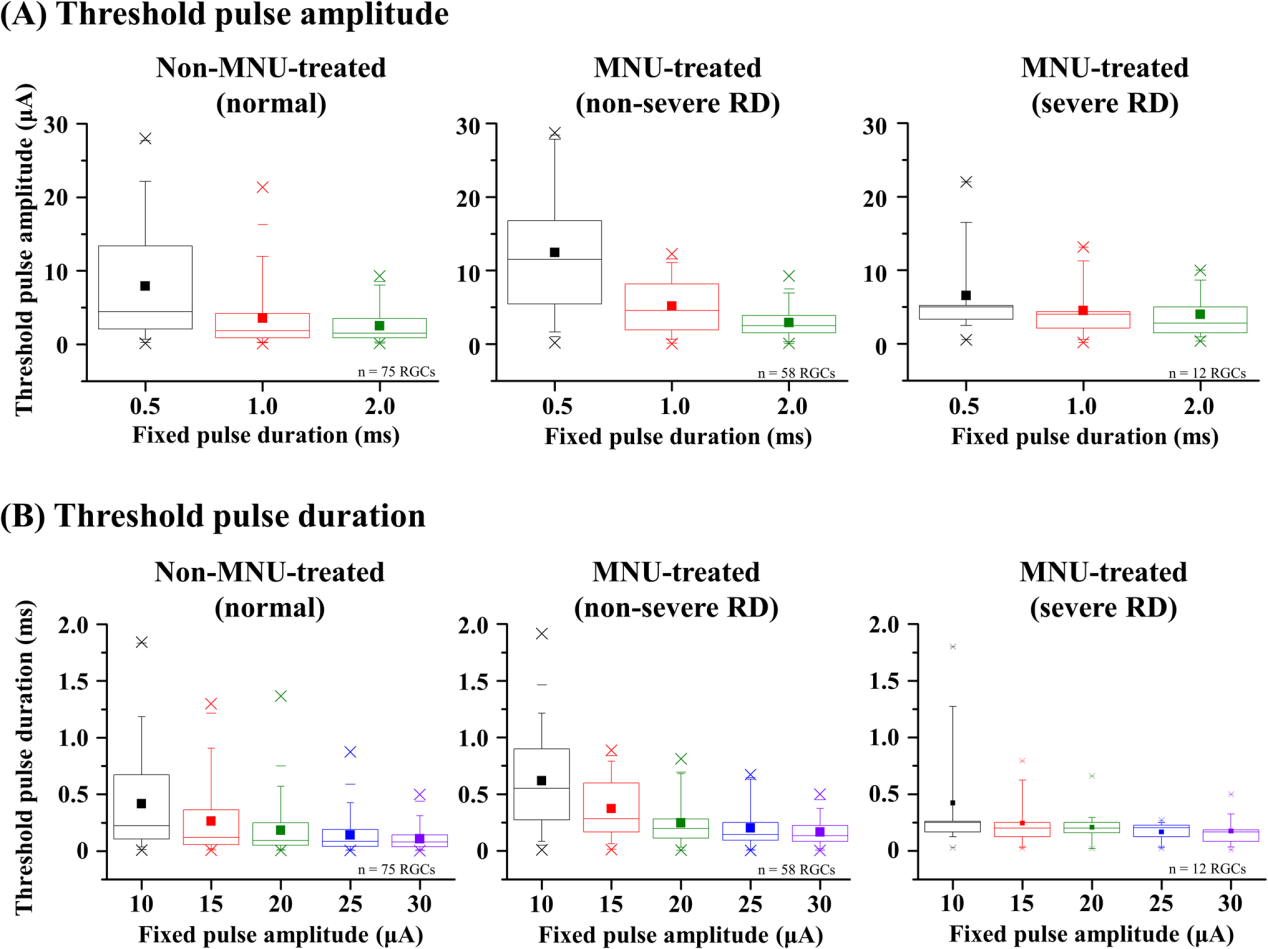
Comparison of threshold pulse amplitude and threshold pulse duration in non-MNU-treated and MNU-treated groups. Values of threshold (**A**) pulse amplitude and (**B**) pulse duration calculated from individual RGCs. The boxes show the range from first to third quartiles. The bottom whisker span from the first quartile box down to 5% and the upper whisker span from the third quartile box up to 95%. The midline of the box represents the median value of thresholds. The filled square symbol represents average value of thresholds. The thin horizontal bars represent 1% and 99%. The minimum and maximum value of threshold are plotted as cross symbols.

Next, we obtained thresholds for pulse amplitude and pulse duration that evoked spikes in well-modulated RGCs (Fig. 5). In all PAM protocols (0.5, 1, and 2 ms fixed pulse), the RD groups in all three degrees of severity showed decreased thresholds of pulse amplitude with an increment of fixed pulse duration. In addition, in all PDM stimulations, all three RD groups showed decreases in thresholds for pulse durations with an increment of fixed pulse amplitude. The obtained threshold values and threshold charge densities are described in Table 1.

**Table 1:** Threshold values (mean ± SEM).

Group		Pulse amplitude modulation			Pulse duration modulation					
		0.5 ms fixed	1 ms fixed	2 ms fixed		10 μA fixed	15 μA fixed	20 μA fixed	25 μA fixed	30 μA fixed
Normal	Threshold current (μA)	7.93 ± 0.88	3.54 ± 0.49	2.52 ± 0.27	Threshold duration (ms)	0.42 ± 0.05	0.26 ± 0.04	0.18 ± 0.03	0.14 ± 0.02	0.11 ± 0.01
	Threshold charge density (mC·cm^−2^·phase^−1^)	0.56 ± 0.06	0.50 ± 0.06	0.71 ± 0.08	Threshold charge density (mC·cm^−2^·phase^−1^)	0.59 ± 0.07	0.56 ± 0.07	0.52 ± 0.07	0.50 ± 0.06	0.46 ± 0.05
Non-severe RD	Threshold current (μA)	12.42 ± 1.06	5.17 ± 0.48	2.92 ± 0.27	Threshold duration (ms)	0.62 ± 0.05	0.37 ± 0.03	0.25 ± 0.03	0.20 ± 0.02	0.17 ± 0.02
	Threshold charge density (mC·cm^−2^·phase^−1^)	0.88 ± 0.07	0.73 ± 0.06	0.83 ± 0.08	Threshold charge density (mC·cm^−2^·phase^−1^)	0.87 ± 0.07	0.79 ± 0.07	0.70 ± 0.08	0.72 ± 0.08	0.70 ± 0.06

### Strength-duration (SD) curve from well-modulated RGCs

**Figure 6:**
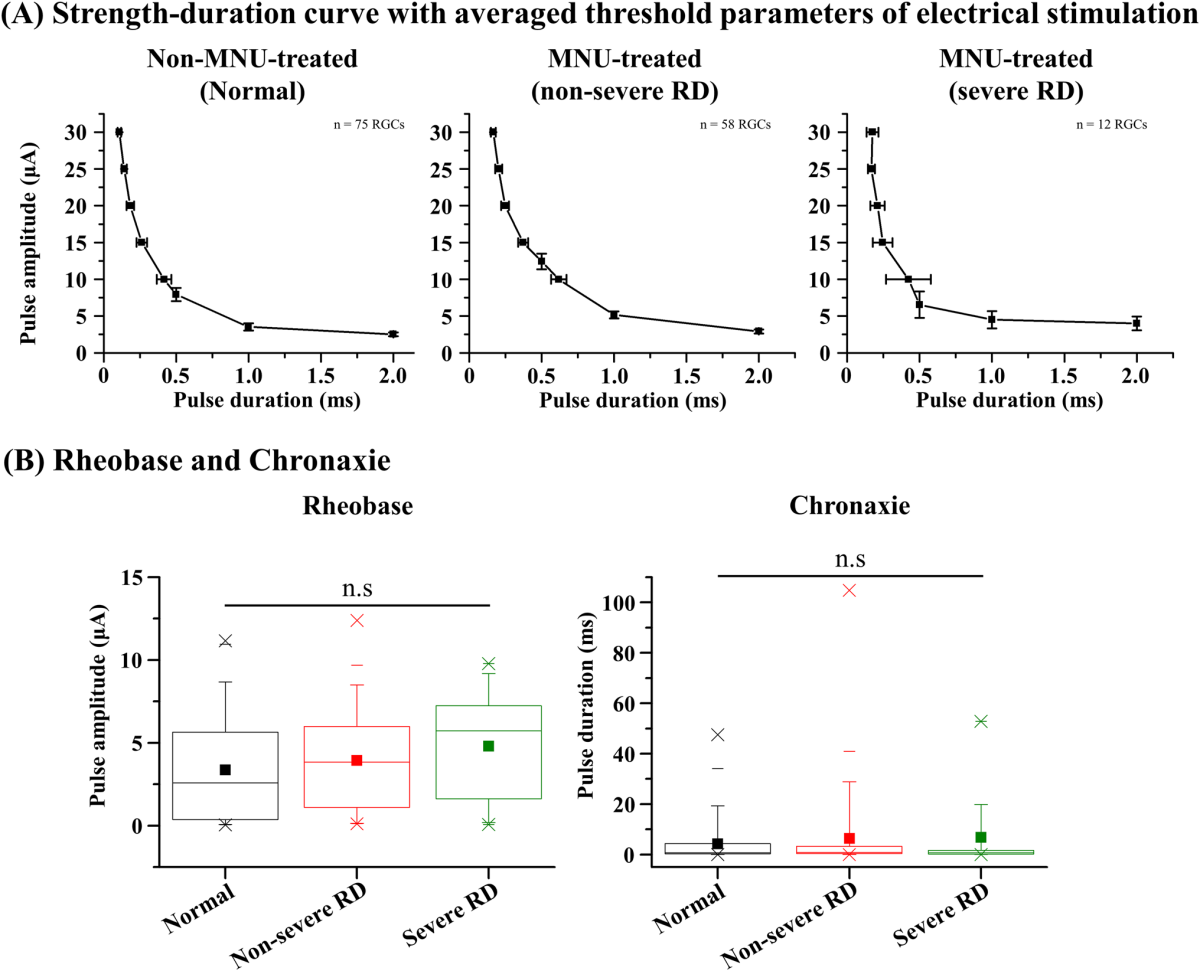
Strength-duration (SD) curve. (**A**) Averaged SD curves of well-modulated RGCs from three different groups. (**B**) Rheobase (left) and chronaxie (right) values calculated from individual well-modulated RGCs. Each SD curve is fitted with hyperbolic functions. Statistical significance among the three groups analyzed using ANOVA (^n.s^p > 0.05).

We constructed a strength-duration curve of well-modulated RGCs. The SD curve was fitted with a hyperbolic function (I = R(1 + C/D), I = amplitude, D = duration, R = rheobase, and C = chronaxie; Fig. 6A). Rheobase and chronaxie were extracted from the fitted curves. The average SD curves seemed to shift upward and to the right as the severity of RD increased; however, there were no noticeable differences in rheobase and chronaxie in the three groups (Fig. 6B). The average values of rheobase were 3.37 ± 0.36, 3.94 ± 0.40, and 4.80 ± 1.05 μA (mean ± SEM) in the normal, non-severe RD, and severe RD groups, respectively. The average values of chronaxie were 4.21 ± 0.95, 6.32 ± 2.11, and 6.84 ± 4.48 ms (mean ± SEM) in the normal, non-severe RD, and severe RD groups, respectively.

## Discussion

In this study, we successfully demonstrated that an MNU-induced RD pig model can be used to optimize parameters for electrical stimulation to further help develop retinal prosthetics.

We previously showed that our new RD model could be successfully induced with MNU in a larger animal^28^. In that study, the results were evaluated by multimodal examinations, including spectral-domain optical coherence tomography (OCT) imaging, ffERG recording, and immunohistochemistry. The MNU-induced RD pig model up to moderate RD mimicked human RP patients well in terms of rod dominant ERG abnormality which shows a decrease of ERG amplitude and delayed implicit time of a and b waves in scotopic condition. However, in the severe RD model, there is a mismatch between the animal model and human RP patients, in terms of RGC death in the central retina. This unexpected RGC death observed in the animal model might be coming from the local heterogeneities in RD expression in the porcine model. Since our previous evaluations were mostly based on the morphological evidence observed in the outer layer of whole retinas and the global response of the retina via ffERG recording, we need to look into the electrophysiological activities of RGCs that are essential for restoring vision in patients with RD using retinal prosthetics.

Moreover, the in-vitro MEA recording with isolated retina is easy to handle in comparison with the in-vivo recording as EEP signal with the living animal. This benefit of the in-vitro recording facilitates the repetition of the experiment. By repeating experiments, we can generate more reliable data, which is one of the strengths of in-vitro study over in-vivo study. Thus, we tried to find the electrical stimulus parameters such as threshold charge density using the in-vitro MEA recording setup.

The in-vitro study detects the RGC response at the retina to electrical stimulation while in-vivo EEP recording detects the visual percepts at the visual cortex. With this difference, the current threshold for RGC spikes or EEP signal should be different.

In this in-vitro study, the threshold current of 8 μA and the threshold charge of 4 nC in wild-type pig retinas are derived. On the other hand, Laube et al. showed the threshold current of 50 μA and the threshold charge of 10 nC for in-vivo EEP recordings in minipigs under epiretinal stimulation^30^. However, our threshold charge density and Laube’s are compatible, respectively 0.58 mC/cm^2^ and 0.26 mC/cm^2^. To our best knowledge, no report for EEP recording can be found using the porcine RD model. Therefore, no comparison is possible between in-vitro recording and in-vivo recording in the porcine RD model. According to literature analysis based on various animal models except for the porcine RD model, there is no difference between threshold charge density with in-vitro recording in the retina and in-vivo recording in the cortex^19^. Thus, finding electrical stimulation parameters such as threshold charge density using an in-vitro setup is meaningful and provides a guideline for in-vivo studies or clinical trials.

Clinically used retinal implants for decades, such as the Argus II and Alpha IMS, are known to stimulate RGCs locally, covering only the macular area of the retina. Because the sizes of the electrode array in the Argus II and Alpha IMS (3.8 mm × 6.7 mm and 3 mm × 3 mm, respectively)^8,31,32^ and that of MEA used in this study (2 mm × 2 mm) are relatively compatible, our findings can be especially useful to estimate RGC responses to retinal prosthetic devices that will be implanted in the retinas of patients with RD*.*

We observed that the MNU-induced RD porcine model followed progressive photoreceptor death, similar to that observed in the genetically induced RD model^33^. Although the degeneration process observed in the MNU-treated model was similar to that of genetically induced ones, there were differences in the distribution of degeneration in the retina. Unlike genetically induced RD, which causes global homogeneous expression of RD throughout the retina, MNU-treated RD exhibited local heterogeneities in RD expression even when MNU was spread evenly throughout the retina^28,34^.

Because of this inconsistency in the expression of RD, the retinal patches that we recorded showed varied severity in degeneration, which may have been neglected in our previous study that used the average of amplitude changes in the ffERG recording^28^. Therefore, to assess the severity of RD more accurately (Fig. 2), we classified recorded neurons into two different RD groups based on the presence of LS-responsive RGCs as severe and non-severe RD groups. We found that the number of ES-responsive RGCs in the non-severe RD group was similar to that in the normal group, whereas the number of ES-responsive RGCs in the severe RD group decreased to about half of the numbers in the non-severe RD group.

Further, the RGC responses evoked by electrical stimulation can be categorized into two groups: direct and indirect. The responses elicited by the direct excitation of electrical stimulation on RGCs occur within 10 ms after the stimulus, whereas the indirect responses are observed from 10 to 100 ms after the stimulus onset, presumably because of the time taken during synaptic transmission from photoreceptors or BCs to RGCs^23,35^. As mentioned in the “Methods” section, since we only investigated ES-responsiveness of indirect response, the reason for the reduced number of ES-responsive RGCs in the severe RD group may be the functional loss of BCs. Histological evidence from our previous study showing destruction in the inner nuclear layer (INL) in the retinas of severe MNU-treated RD pigs supports this hypothesis^28^.

Furthermore, we observed that the ES-responsive RGCs in MNU-treated retinas showed less ES-responsiveness than normal retinas of pigs (Figs. 4, 5). Specifically, the ES-responsiveness of MNU-treated retinas was saturated by relatively weaker electrical stimulation than that of normal retinas. This means that there is a limit to evoking RGC responses regardless of the intensity of electrical stimulation. In other words, stronger electrical stimulation will not be effective in RD retinas once it passes the point where the RGC responses start to saturate.

We were able to stimulate RGCs with following threshold charge density (normal: 0.55 ± 0.07 mC cm^−2^ phase^−1^; non-severe group: average charge density = 0.78 ± 0.08 mC cm^−2^ phase^−1^, calculated from the data in Table 1). In comparison with a lower animal model, the RGCs in the normal and RD porcine retina required higher threshold charge density (for instance, WT mice required 0.35 ± 0.05 mC cm^−2^ phase^−1^ and *rd1* mice required 0.41 ± 0.08 mC cm^−2^ phase^−1^, calculated from our previous study^11^). However, because the amount of electrical charge density measured in this study was less than the safety limits found in earlier studies^19,21^, the degenerated retina of the porcine model could respond with a relatively safer range of electrical stimulation. This is an important finding that should be considered when developing neural prosthetic devices.

The overall thresholds for amplitude and duration to evoke RGC responses in the MNU-treated retinas were higher than those in normal retinas of pigs (Table 1). These results have been observed in other animal models of RD. In our previous study, we reported that the RGCs in the completely degenerated retinas of *rd1* mice required higher intensities of electrical stimulation to elicit responses than the wild-type RGCs of C57BL/6J mice^11^. Moreover, phosphenes in human patients with RP are less induced by electrical stimulation than that in sighted human subjects^36,37^. Collectively, these results can help optimize parameters for electrical stimulation in patients with RD in the future.

Although it is difficult to identify the exact sources of the reduced ES-responsiveness in RD retinas observed in this study, the following plausible causes are worth considering that can eventually help overcome the technical obstacles that retinal prosthetic devices render:

1. Glia sealing: Gliosis by Müller glial cells appears in degenerated retinal networks after the total death of photoreceptors^38^. It is also known that Müller glial cells can seal both outer nuclear layers and ganglion cell layers. This enclosure of RGCs by glial cells has been also reported in all cases of genetically induced RD, MNU-induced RD animal models, and human patients with RP^28,34,39–41^. This sealing effect on RGCs may have prevented the surviving neurons from being stimulated by electrical pulses in the degenerated retina in the present study.
2. Retinal remodeling: In the degenerated retinal circuit, surviving neurons tend to develop abnormal synapses to compensate for lost connections by deafferentation and prevent further cell death. Based on this reasoning, Marc et al. suggested that wiring errors among surviving neurons (BC, AC, and RGC) can induce abnormal changes in the membrane potential of BC by excitatory inputs from presynaptic neurons^5^. These abnormalities in membrane potential may have contributed to the reduced responsiveness of RGCs to electrical stimulation.

No significant differences were observed among the chronaxie and rheobase values in all three groups (Fig. 6). This finding implies that the electrical properties of RGCs, even in the severe RD groups, are well preserved regardless of the severities of RD, thereby maintaining electrical responsiveness. This resilience of RGCs makes them possible to be evoked by electrical stimulation using a retinal prosthesis. Sekirnjak et al. studied chronaxies and rheobase of normal RGCs in three different animals, namely monkeys, guinea pigs, and rats^19^. They reported similar patterns in the SD relationship regardless of the species. However, their averaged values of chronaxie and rheobase (338 ± 81 μs and 0.60 ± 0.11 μA) were much lower than those found in this study (4210 ± 950 μs and 3.37 ± 0.36 μA). This discrepancy may be due to the difference in origins of the RGC spikes. Sekirnjak et al. mainly investigated direct responses, whereas we focused on indirect responses. Several previous studies have reported that BCs indeed require a longer pulse duration than RGCs^14,42^.

In conclusion, our results suggest that the degenerative porcine retina requires a higher charge than the normal porcine retina. In addition, in the early stage of RD, it is easy to induce RGC spikes through electrical stimulation using a retinal prosthesis; however, when the degeneration progresses to a severe degree, recovering the patient’s vision will be relatively difficult.

## Methods

### Animals

All procedures followed the guidelines of the Association for Research in Vision and Ophthalmology Statement for the Use of Animals in Ophthalmic and Vision Research. This study was approved by the Institutional Animal Care and Use Committee of the Korea University College of Medicine (approval number: KOREA-2018-0002-C1). All our in-vivo and in-vitro experimental protocols were carried out in compliance with the ARRIVE guidelines [the Institutional Animal Care and Use Committee of the Korea University College of Medicine (approval number: KOREA-2018-0002-C1)]. We used nine eyes from nine female mini-pigs (MICROPIG, APURES Co., Ltd, Pyeongtaek-si, Gyeonggi-do, Korea) in this study. The mean age of mini-pigs was 11.13 ± 2.31 months, and the mean body weight was 29.86 ± 2.94 kg. RD was induced in five eyes by intravitreal loading of MNU, which induces apoptosis in photoreceptors. The other four eyes were used as controls. The details are provided in our previous publication^28^.

### Retinal preparation

The mini-pigs were anesthetized by an intravenous injection of alfaxalone (1 mg/kg Alfaxan; Vetoquinol UK, Towcester, Northamptonshire, UK) into the marginal auricular vein following premedication, which comprised a subcutaneous injection of atropine (0.05 mg/kg), intramuscular injection of xylazine (1 mg/kg Rompun; Bayer Corp., Shawnee Mission, KA, USA), and azaperone (4 mg/kg Stresnil; Mallinckrodt Veterinary Inc., Indianapolis, IN, USA). The subjects were euthanized immediately after enucleation of the eyeball. The retina was isolated and cut into patches of approximately 3 mm × 3 mm from a visual streak (Fig. 1A). The detailed procedures for the preparation have been described in previous studies^22,43^. The retinal patch was prepared under near-infrared illumination in an artificial cerebrospinal fluid (ACSF) solution (124 mM NaCl, 10 mM glucose, 1.15 mM KH_2_PO_4_, 25 mM NaHCO_3_, 1.15 mM MgSO_4_, 2.5 mM CaCl_2_, and 5 mM KCl; all from Sigma-Aldrich, St. Louis, MO, USA) bubbled with 95% O_2_ and 5% CO_2_ to maintain a pH of 7.3–7.4 at 25 °C. The isolated retina was mounted with the RGC layer down on a perforated MEA (pMEA, 60pMEA200/30iR), and continuously perfused with the oxygenated solution using a peristaltic perfusion system (PPS2; Multichannel Systems GmbH, Reutlingen, Germany) during the experiment (Fig. 1B).

### Data recording system

The RGC spikes were recorded using a 60-channel MEA. Briefly, the data acquisition system (MEA2100-Lite-system; Multichannel Systems GmbH, Reutlingen, Germany) included a planar 60-channel array, an amplifier (MEA2100-60-headstage), an MCS-Lite interface board, and temperature control units (TC02 and PH01). The retinal activities were recorded with a bandpass from 1 to 3000 Hz; a gain of 1200; and a sampling rate of 25 kHz. The electrode array had 59 titanium nitride (TiN) active electrodes in an 8 × 8 grid layout on a porous polyimide foil isolator with an electrode diameter of 30 μm, inter-electrode distance of 200 μm, and a large internal reference electrode as channel 15. The impedance level was approximately 50 kΩ at 1 kHz. The amplifier was placed in a Faraday cage connected to a laboratory-made ground system. The raw trace was recorded using the online data acquisition software of the Multi Channel Suite package (Multi Channel Experimenter version 2.4.4; Multichannel Systems GmbH, Reutlingen, Germany).

### Light stimulation

The light stimuli were generated by custom-made software, written in MATLAB with Psychtoolbox^44,45^ and presented via a liquid–crystal display (LCD) projector (AJD-36; OMAX, Bucheon-si, Gyeonggi-do, South Korea). We applied white full-field illumination (Light ON; intensity: 10.15 μW/cm^2^) for 2 s and black background light (Light OFF; intensity 0.38 μW/cm^2^) for 2 s alternatively. The ON and OFF cycles were sequentially repeated 20 times (Fig. 1C). The abovementioned light intensity corresponding to photopic vision (10.15 μW/cm^2^) was measured using a calibrated radiometer (ILT-5000; International Light Technologies, Peabody, MA, USA).

### Electrical stimulation

Among the 59 electrodes of the pMEA, an electrode at the center was selected for stimulation, and the others were used for recording. Pulse trains were applied to the stimulation electrode using a stimulus generator in the MEA2100-60-headstage. The stimuli consisted of symmetric, cathodic, phase-first biphasic pulses. Trains of 50 identical pulses were applied every 1 s along with an increase in the pulse amplitude (10, 15, 20, 25, and 30 μA) and pulse duration (500, 1000, and 2000 μs) (Fig. 1C).

### Data analysis

From the raw waveform of the retinal recording, the RGC spikes were isolated using a 100 Hz high-pass filter with the offline data analysis software of the abovementioned Multi Channel Suite package (Multi Channel Analyzer; Multichannel Systems GmbH, Reutlingen, Germany). The threshold for spike detection was set to four times the standard deviation of the background noise. The recording data were processed with a spike sorting software (Offline Sorter™; Plexon Inc., Dallas, TX, USA) to separate multiunit activities containing different spike waveforms into individual cell units using principal component analysis. Data analysis was performed using a commercial analysis software (NeuroExplorer^®^; Nex Technologies, Colorado Springs, CO, USA) and a custom-made MATLAB (MathWorks, Natick, MA, USA) code.

The RGC responses evoked by electrical stimulation were defined as the relative changes in the number of RGC spikes after the stimulation onset. To quantify the spontaneous RGC firings, we calculated the average number of spikes before the stimulation onset in the 20 bins (bin = 100 ms). Next, we calculated the average number of spikes in 20 trials of electrical stimulation within the post-stimulus period from 10 to 100 ms. We disregarded the signals from 0 to 10 ms to remove the stimulus artifact and direct RGC responses. The threshold level was defined as 1.5 times the average value of spontaneous activities in the 20 trials. Statistical analysis was performed using a commercial statistical software (IBM SPSS Statistics 24; International Business Machines Corporation, IBM)., New York, NY, USA).

## Supplementary Information

Supplementary Figure S1.

## Data Availability

The datasets generated during and/or analyzed during the current study are available from the corresponding authors on reasonable request.
