# Supplementary figures and images for: Electrical response of retinal ganglion cells in an N-methyl-N-nitrosourea-induced retinal degeneration porcine model
Source: Sci Rep. 2021 Dec 17;11:24135. doi: 10.1038/s41598-021-03439-w (PMC8683404; doi:10.1038/s41598-021-03439-w)

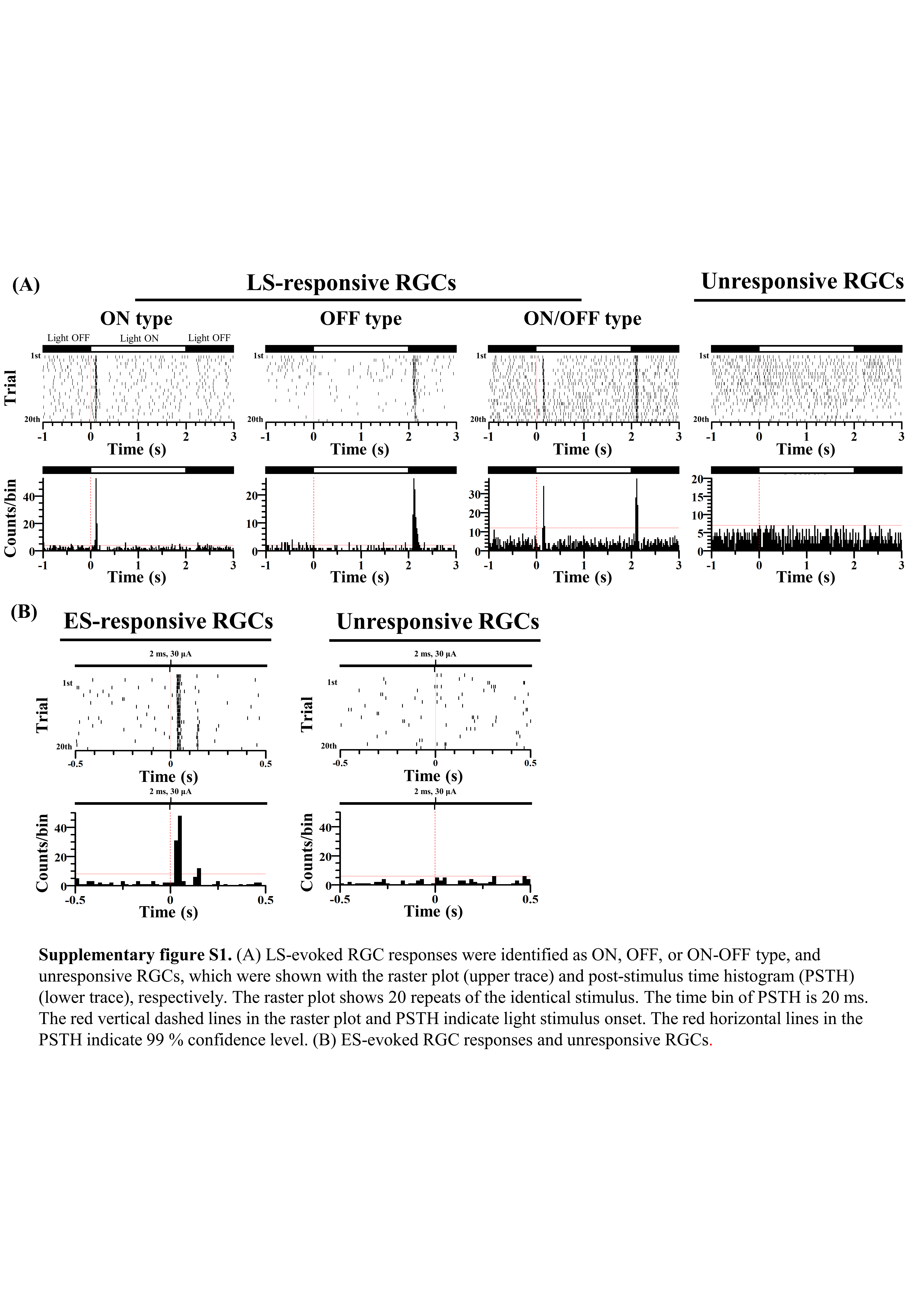

Supplement: Supplementary file 1 — Supplementary Figure S1. [file 41598_2021_3439_MOESM1_ESM.gif]
